# The neurobiological foundation of effective repetitive transcranial magnetic brain stimulation in Alzheimer's disease

**DOI:** 10.1002/alz.70337

**Published:** 2025-06-18

**Authors:** Annibale Antonioni, Alessandro Martorana, Emiliano Santarnecchi, Harald Hampel, Giacomo Koch

**Affiliations:** ^1^ Department of Neuroscience and Rehabilitation University of Ferrara Ferrara Italy; ^2^ Memory Clinic, Department of Systems Medicine University of Tor Vergata Rome Italy; ^3^ Gordon Center for Medical Imaging, Mass General Research Institute, Harvard Medical School Boston Massachusetts USA; ^4^ Sorbonne University, Alzheimer Precision Medicine (APM), AP‐HP, Pitié‐Salpêtrière Hospital, 47‐83 Bd de l'Hôpital Paris France; ^5^ Experimental Neuropsychophysiology Lab, Department of Clinical and Behavioural Neurology Santa Lucia Foundation IRCCS Rome Italy; ^6^ Center for Translational Neurophysiology of Speech and Communication (CTNSC) Italian Institute of Technology (IIT) Ferrara Italy

**Keywords:** Alzheimer's disease (AD), animal models, noninvasive brain stimulation (NIBS), pathological cascade, plasticity, preclinical research, repetitive transcranial magnetic stimulation (rTMS), transcranial magnetic stimulation (TMS)

## Abstract

Noninvasive brain stimulation (NIBS) techniques, such as repetitive transcranial magnetic stimulation (rTMS), are promising candidate therapeutics for Alzheimer's disease (AD). We review the evidence supporting the fundamental mechanisms of action of rTMS treatments in AD. rTMS exerts profound effects at different neurobiological and systems neurophysiological levels. By engaging distinct pre‐ and postsynaptic structures within the stimulated neural network, it directly or indirectly influences various cellular and molecular components. In AD, rTMS influences synaptic plasticity, inducing lasting structural changes and broad reorganization of functional and structural connectivity at the macroscale level. Importantly, it modulates neurotransmitter circuits characteristically disrupted in AD and restores the excitation/inhibition balance by targeting glutamatergic and γ‐aminobutyric acid (GABA)ergic pathways. Moreover, rTMS increases neurotrophic factors, counteracts amyloid and tau accumulation, and mitigates neuroinflammation by reducing microglial activation and pro‐inflammatory cytokines release. Therefore, maturing preclinical evidence could guide future precision medicine therapeutic strategies based on personalized NIBS in AD patients.

**Highlights:**

- Noninvasive brain stimulation (NIBS) techniques, such as repetitive transcranial magnetic stimulation (rTMS), are promising candidate therapeutics for Alzheimer's disease (AD).
- rTMS modulates neuroplasticity, neurotransmission, and neuroinflammation.
- Preclinical research shows disease‐specific neurobiological effects of rTMS in AD.
- Promising data from AD patients suggest the translatability of animal model results.
- Preclinical data may guide precision medicine strategies through personalized NIBS.

## 1 BACKGROUND

Alzheimer's disease (AD) is a complex nonlinear progressive brain proteinopathy, leading to primary neurodegeneration in distinct areas of the brain. The late clinical stage of AD is typically characterized by cognitive decline and dementia with increasing impairment of activities of daily living and loss of functional independence.^1^ Despite decades of scientific and economic efforts, the etiology remains only partly understood and characterized, due to the decade‐long complex and multifaceted pathophysiology.^2^ AD is associated with a pathologically altered amyloid‐beta (Aβ) pathway with the accumulation of soluble neurotoxic species that aggregate into extracellular Aβ plaques.^3^ Another core aspect of AD pathophysiology evolves through hyperphosphorylation of intracellular tau‐protein (pTau), spreading and production of toxic species, leading to the formation of paired helical filaments (PHF) and ultimately aggregated neurofibrillary tangles (NFTs) associated with regional neurodegeneration and disease progression.^4^ Furthermore, multiple, intersecting molecular and cellular mechanisms, such as neuroimmune and inflammatory events,^5^ mitochondrial dysfunction, oxidative stress, disruptions in neurovascular integrity, and cellular mechanisms involved in brain protein homeostasis, form the basis for phenomenological‐clinical disease heterogeneity and are involved in the long and complex AD pathophysiology.^6^, ^7^, ^8^

Together, these findings reflect an intricate, multifactorial pathophysiology emphasizing the need for a broad and comprehensive approach to therapeutic development for AD, supported by inclusive, unbiased hypothesis‐independent biomarker‐guided classification and pathology and pathophysiology detection systems, such as the AT(X)N framework for AD.^9^

Importantly, damage and disruption to synaptic plasticity, which is defined as the ability of synapses to strengthen or weaken over time in response to increases or decreases in their activity, has increasingly been recognized as a hallmark of AD. Thus, targeting and improving synaptic plasticity represents an attractive therapeutic strategy.^10^, ^11^, ^12^, ^13^ In this perspective, noninvasive brain stimulation (NIBS) techniques are emerging as novel personalized therapeutics in AD.^12^, ^14^ NIBS techniques enable the modulation of neuronal populations by applying electrical (transcranial electrical stimulation) or magnetic (transcranial magnetic stimulation [TMS]) stimuli to the scalp.^15^ This approach can influence neuronal activity not only at the sites of stimulation but also in remote, interconnected brain regions via network effects.^16^ While single‐pulse TMS techniques primarily induce a transient depolarization of neurons beneath the coil through Faraday's principle of electromagnetic induction, repetitive TMS (rTMS) has been shown to produce enduring neuronal changes that outlast the stimulation period.^17^ These changes include both long‐term potentiation (LTP) and depression (LTD) of activity, likely through modulation of complex mechanisms involving calcium‐dependent processes and glutamate receptors—the principal excitatory neurotransmitter in the central nervous system (CNS).^18^ Specifically, high‐frequency rTMS (HF‐rTMS, > 5 Hz) leads to an increase in cortical excitability through LTP‐like mechanisms, whereas low‐frequency rTMS (LF‐rTMS, < 1 Hz) is associated with its decrease because of LTD‐like mechanisms. The impairment of LTP‐like cortical plasticity has been recently identified as one of the key neurophysiological features in AD.^19^ Hence, rTMS might be an ideal tool to restore altered LTP and promote functional rearrangements of connectivity.^12^ Moreover, rTMS is suitable for delivering personalized therapy based on a precision medicine approach. The combination of neuro‐navigated TMS with electroencephalography (TMS‐EEG)^20^, ^21^ allows for a direct probe of local and widespread cortical dynamics through the recording of TMS‐evoked potentials.^22^ By applying a series of magnetic pulses over a target area, it is possible to map how the neural signal propagates from the site of stimulation within the network in each patient.^20^, ^21^ Functional and structural brain magnetic resonance imaging (MRI) can be used to integrate these neurophysiological signals within the individual anatomical and functional network.^23^

Given that rTMS is cost‐effective and relatively easy to perform and can lead to a durable and specific modulation of neuronal activity in a safe manner in most of the general population, interest in its therapeutic applications is steadily rising. Consistently, it has already shown considerable potential in a range of neurological and psychiatric disorders.^24^, ^25^ The neurobiological mechanisms underlying its efficacy are under active investigation, and much of the available evidence stems from animal models. These models provide critical insights that would be challenging to explore ethically in humans. Indeed, animal models are essential in clarifying the cellular and molecular underpinnings of rTMS to inform clinical applications.^26^

While several prior reviews have addressed the therapeutic potential of rTMS in AD, most of these works predominantly focus on clinical efficacy, stimulation parameters, or target selection in human patients, often providing an overview of clinical trial outcomes.^12^, ^27^, ^28^, ^29^ Moreover, although some earlier studies have indeed explored the neuroprotective potential of rTMS in neurological and psychiatric diseases by examining neurobiological findings from preclinical models, these studies are generally brief, exploratory reviews.^30^, ^31^ Thus, a comprehensive synthesis exploring the neurobiological underpinnings of rTMS efficacy in AD, from animal models to translational evidence in clinical populations, is missing. Given the rapidly growing body of research in this field and the promising implications for developing new therapeutic strategies for AD patients, an up‐to‐date, integrative overview addressing these gaps seems both timely and essential. Here, we delve into how rTMS modulates specific molecular, cellular, and synaptic processes that are critically involved in the pathophysiology of AD. By adopting a mechanistic lens, we seek to go beyond summarizing outcomes and instead offer an interpretative framework that connects specific neurobiological effects of rTMS to the pathological hallmarks of AD. Furthermore, we emphasize the translational value of these findings by outlining how preclinical evidence can inform future precision medicine approaches for NIBS application in AD, supporting the design of more targeted and biologically informed clinical interventions.

## 2 EVIDENCE ABOUT THE NEUROBIOLOGICAL EFFECTS OF RTMS IN HEALTHY ANIMAL MODELS

TMS can induce a wide range of neurobiological effects. Beyond activating distinct pre‐ and postsynaptic structures within the stimulated neural network, TMS may exert direct or indirect effects on various cellular and molecular components, including mitochondria, glial cells, calcium stores and buffers, translation and transcription factors, postsynaptic scaffolds, adhesion molecules, and ligand‐ or voltage‐gated channels and metabotropic receptors.^32^ Importantly, much of the evidence arises from studies on animal models. For instance, TMS induced or modulated synaptic plasticity in both healthy rodents and aging rats.^33^, ^34^ Experimental studies in rodents showed that rTMS leads to complex neurobiochemical effects, including the expression of immediate early genes (IEGs, such as *c‐Fos, c‐Myc, c‐Jun, zif268*, and activity‐regulated cytoskeleton‐associated protein *[Arc]*), modulation of neurotransmitter release (including dopamine, acetylcholine, serotonin, gamma‐aminobutyric acid [GABA], glutamate, and glycine), alterations in α‐amino‐3‐hydroxy‐5‐methyl‐4‐isoxazolepropionic acid (AMPA) and N‐methyl‐D‐aspartate (NMDA) glutamate receptor dynamics (which affect calcium ion dynamics), influences on neuroendocrine axis, regulation of cell hydration, neuroprotective actions aimed at mitigating oxidative stress and inflammation, and intense stimulation of neurotrophic factors.^35^, ^36^, ^37^, ^38^, ^39^, ^40^, ^41^ Even a single session of rTMS may produce significant effects in the rat brain, particularly an increase in serotonin and its metabolites and a change in the properties of serotonergic receptors.^42^, ^43^ However, it is not always clear whether these effects are due to rTMS or the stress associated with the experimental protocol in the tested animals.^44^ For example, in rats, a single session of both LF‐ (1 Hz) and HF‐rTMS (10 Hz) enhanced *c‐Fos* expression in all examined cortical areas.^45^ On the other hand, intriguingly, only a specific type of rTMS, known as intermittent theta burst stimulation (iTBS), which is recognized for inducing LTP‐like effects, increased both *c‐Fos* and *zif268*, a gene essential for the induction and persistence of LTP, in several brain regions.^45^, ^46^, ^47^

Consistently, a study comparing LF‐rTMS (1 Hz), iTBS, and continuous TBS (cTBS, a protocol that induces LTD‐like effects) demonstrated that all of them influence the GABAergic circuitry of the rat cortex but in a protocol‐specific manner.^48^ These differences may be attributed to the distinct gene expression patterns. Indeed, different studies have observed increased *c‐fos* expression only in specific brain areas after an acute single rTMS train, while others have documented its widespread increase after chronic rTMS stimulation in the rat cortex and hippocampus.^49^, ^50^

Notably, rTMS protocols, at least in some cases, might require multiple sessions to fully develop their potential from a neurobiological perspective. In this regard, chronic HF‐rTMS (20 Hz, 11 weeks) treatments do not lead to detrimental structural changes in the CNS, as no increases in glial fibrillary acidic protein (GFAP) levels are detectable in rats following this protocol.^51^ Moreover, long‐term rTMS does not induce cognitive impairments indicative of neuronal damage in vivo. On the contrary, the same research suggests a neuroprotective role, as rTMS enhances neuronal viability and counteracts oxidative stressors, such as Aβ and glutamate toxicity, in vitro.^51^ These findings further support the safety profile of chronic rTMS from both a structural and functional perspective. Thus, even long‐term treatments have neuroprotective effects, and there is no damage because of repeated stimulation. Moreover, chronic LF‐rTMS treatment (0.5 Hz) modulates the concentration of both GABA and glutamate in the hippocampus, striatum, and hypothalamus of rats.^52^ This suggests that rTMS can not only modulate the excitation/inhibition balance, which is crucial for proper CNS functioning, but also induce effects in deep brain regions that are not directly accessible by the coil. These effects likely arise from rTMS's ability to engage complex cortico‐subcortical networks through transsynaptic and polysynaptic pathways, leading to downstream modulation of deep structures functionally connected to the stimulated cortical targets.^53^ This property is particularly relevant for clinical applications, as it implies that rTMS protocols can influence key regions involved in AD pathophysiology, such as the hippocampus, basal forebrain, and thalamus, despite their anatomical inaccessibility to direct stimulation.^54^, ^55^ Understanding and optimizing these network‐level effects may be crucial for developing more effective and targeted therapeutic strategies.

Furthermore, chronic rTMS treatment modulates cortical serotonergic, adrenergic, and dopaminergic circuits in the brains of rats.^44^, ^56^ Therefore, rTMS could be a relevant tool to regulate multiple neurotransmitter networks, underscoring its potential in modulating a wide range of brain functions. Importantly, at least some of the neurobiological effects of chronic HF‐rTMS (5 Hz) result from a specific modulation of the expression of cyclic adenosine monophosphate (cAMP) response element‐binding protein (CREB), a cellular transcription factor critically involved in plasticity‐associated signaling pathways.^57^, ^58^, ^59^ Thus, CREB might be a key mediator of synaptic plasticity and learning improvements due to rTMS treatments.^60^

Furthermore, HF‐rTMS induces the expression of the GLUR1 subunit of the AMPA receptors.^61^ This mechanism enables the recruitment of a sufficient number of AMPA receptors to trigger the opening of NMDA channels, allowing an influx of Ca^2+^ ions that activate specific calcium‐sensitive cellular pathways, leading both to feedback on AMPA receptors—including the synthesis of new receptors and phosphorylation of existing ones—and to the initiation of long‐term processes that strengthen synaptic connections through modifications at both pre‐ and postsynaptic sites.^62^ Indeed, synaptic plasticity, involving both structural and functional changes at the synaptic level, is essential in learning.^63^ Consistently, NMDA receptors are crucial for hippocampal LTP processes involved in memory formation; accordingly, their blockage or downregulation results in memory impairments.^64^ However, excessive glutamate release, known as excitotoxicity, can lead to the dysregulated opening of ionophoric channels, such as NMDA channels, allowing excessive Ca^2^^+^ influx, which is believed to be instrumental in neurodegenerative diseases.^65^ Indeed, calcium plays a significant role in regulating numerous molecular pathways, but excessive concentrations can trigger a cascade of events that severely disrupt neuronal function.^66^ Importantly, rTMS modulates NMDA receptor activity in a controlled manner, promoting synaptic plasticity without provoking neurotoxic effects. Consistently, rTMS‐induced NMDA activation is tightly regulated by homeostatic mechanisms, including concurrent modulation of GABAergic circuits and neurotrophic factor release, which help maintain a balance between excitation and inhibition.^67^ Moreover, rTMS enhances neuroprotective pathways, further supporting synaptic resilience and preventing excitotoxic damage.

Indeed, HF‐rTMS could enhance dendritic complexity and neuronal spine number through brain‐derived neurotrophic factor (BDNF) and Ca^2+^‐dependent signaling pathways in the mouse's primary motor cortex.^68^ Specifically, NMDA‐dependent aftereffects on synaptic plasticity are also critically dependent on rTMS‐induced modulation of the BDNF‐Tropomyosin receptor kinase B (TrkB) pathway, which could justify the latency, typically 5–10 min, to observe changes following repetitive protocols.^69^, ^70^ Moreover, LF‐rTMS enhances hippocampal endogenous neurotrophins and NMDA receptor expressions.^71^ In particular, the binding between BDNF and its TrkB receptor tyrosine kinase leads to autophosphorylation of TrkB, triggering a transduction cascade including mitogen‐activated protein kinase (MAPK)/extracellular signal‐regulated kinases (ERK)1/2 and phosphatidylinositol‐4,5‐bisphosphate 3‐kinase (PI3K)/ protein kinase B (Akt) pathways in which signals are transferred to the nucleus to activate transcriptional factors and, thus, gene expression.^58^, ^72^ This process could explain the long‐term change in synaptic protein expression due to BDNF‐TrkB pathway activation following rTMS protocols.^69^, ^73^ HF‐rTMS (10 Hz) demonstrated a lasting enhancement of glutamatergic synaptic strength, accompanied by structural remodeling of dendritic spines.^74^ This is crucial when considering that, in addition to NMDA‐ and AMPA‐related mechanisms, the induction of LTP is critically influenced by changes in the number of synaptic spine contacts and the shape of spine heads.^75^, ^76^ Furthermore, rTMS might prevent neuronal death by influencing the B‐cell lymphoma 2 (Bcl‐2) family, particularly those elements that stimulate apoptosis.^77^, ^78^ Indeed, an aged rabbit model demonstrates age‐related increases in susceptibility to oxidative stress and a higher risk of hippocampal neurodegeneration mediated by the Bcl‐2 protein family.^79^ Furthermore, a study on genetically modified mice overexpressing Bcl‐2 demonstrated reduced apoptosis, increased production of new granule cells, and an expanded granule cell layer in the hippocampus.^80^ This aspect is critical, as new granule cells exhibit greater synaptic plasticity and more robust LTP mechanisms than aged ones.^81^ Conversely, studies demonstrate an increased threshold for LTP induction in older memory‐impaired rats compared to both young and adult rats at the perforant path–granule cell synapse.^82^ Given that hippocampal granule cells are crucial for mediating LTP, understanding how to promote their survival has significant therapeutic implications.^83^ Modulating the Bcl‐2 protein family could be crucial in promoting hippocampal neurogenesis, a potentially relevant factor in numerous neurological and psychiatric disorders.^30^, ^31^ This beneficial effect of rTMS is likely to act synergistically with other mechanisms, such as the rTMS‐induced increase in hippocampal BDNF levels, which plays a key role in neuroprotection and cognitive enhancement also due to its ability to increase cholinergic enzymes levels, including acetylcholinesterase (AChE).^84^, ^85^, ^86^, ^87^ Indeed, an HF‐rTMS protocol (5 Hz) demonstrated enhanced synaptic plasticity through increased levels of BDNF, NMDA receptor subtype 2B (NR2B, a subunit of the post‐synaptic glutamate NMDA receptor), and synaptophysin (SYP) in the hippocampus of adult rats, resulting in improved spatial learning and memory.^88^

Taken together, these findings suggest that rTMS might play a crucial role in preserving and modulating structural synaptic plasticity within the hippocampus of aging mice, specifically restoring impaired hippocampal LTP‐like mechanisms through various neurobiological processes.^89^ Therefore, the therapeutic potential of these modifications has been explored in disease models, including those of AD. In the following, some animal models of AD commonly used in current research will be briefly described. Afterward, examples of evidence obtained from the application of rTMS in AD animal models will be presented from different perspectives to provide a comprehensive overview of the multiple pathways modulated by this NIBS technique and involved in the pathophysiology of this complex disease.

## 3 A BRIEF OVERVIEW OF COMMON AD ANIMAL MODELS

Animal models of AD enable researchers to replicate, as faithfully and temporally accurately as possible, the pathological sequence of events observed in human disease.^90^ Similar to humans, these models can mimic either sporadic or genetic AD forms. Importantly, mice, even at advanced ages, do not naturally develop Aβ plaques or NFTs and, therefore, cannot serve as natural AD models.^91^ Therefore, rodents must be genetically engineered to overexpress pathogenetic genes, such as amyloid precursor protein (*APP*) and presenilin (*PSEN*) (usually controlled by highly active gene promoters), to enhance Aβ production, aggregation, and deposition rate.^92^, ^93^ Specifically, familial AD (FAD)‐associated mutations in *APP* are located near the β‐site APP cleaving enzyme (BACE) cleavage site (e.g., the Swedish mutation), in critical amino acids that influence its aggregation propensity (e.g., the Arctic and Dutch mutations), or close to the γ‐secretase cleavage site, promoting increased production of the longer Aβ42 peptide (e.g., the London mutation).^94^ Based on this approach, around 20 FAD animal models have been developed.^91^, ^95^ Moreover, these models display specific alterations in hippocampal LTP‐like mechanisms, AMPA receptor surface trafficking, astrocytic signaling, brain inflammation, oxidative stress, and structural modifications to synaptic spine architecture not only in the hippocampus but also at the cortical level.^96^, ^97^, ^98^, ^99^, ^100^, ^101^ However, *APP*‐overexpressing models fail to develop NFTs, though many show detectable pTau,^102^ perhaps because of the inability of human Aβ accumulation to directly trigger NFTs formation. Thus, different models, such as the triple transgenic (3xTg) one, have been developed to study tauopathies based on genetic alterations in the Tau protein, while triggering receptor expressed on myeloid cells 2 (*TREM2*) models have been developed to explore the role of microglia in AD pathogenesis.^103^, ^104^, ^105^, ^106^ While these models are invaluable for modeling multiple aspects of AD neuropathology, they generally fail to fully replicate all neuropathological features observed in human patients. Furthermore, they tend to accumulate toxic products in a temporally distinct manner and/or with a different timeline relative to the onset of cognitive deficits.^91^ Moreover, it is essential to recognize that, in humans, FAD represents only a small proportion compared to sporadic cases.^1^ This distinction is particularly significant given that the causes of familial and sporadic AD cases differ, as do their age of onset (early in FAD and late in sporadic cases) and neuropathological features.^107^, ^108^

To address these differences, animal models have been developed to replicate the anomalies characteristic of sporadic AD cases, focusing on risk factors such as metal ion imbalances and oxidative stress, lipid metabolism abnormalities, neuroinflammation, gut microbiota disorders, autophagy dysfunction, and insulin resistance—factors commonly associated with aging but often underrepresented in FAD models and likely preceding the emergence of tau and Aβ pathologies, at least according to some disease progression models.^108^, ^109^ Examples of these models include the streptozotocin‐induced AD model^110^; the acrolein model, where this toxic agent induces lipid peroxidation associated with NFTs formation and Aβ polymerization^111^; the AAV1‐IPP2A model, which utilizes an inhibitor of protein phosphatase 2A to induce Tau hyperphosphorylation^112^; and models developed using high‐cholesterol diets, senescence‐accelerated mice (SAM), repeated traumatic brain injury (TBI), metallic ion alterations, and oxidative stress induction.^113^, ^114^, ^115^, ^116^ Importantly, the direct CNS injections of Aβ oligomers can lead to significant cognitive impairments in rodent behavioral tasks.^117^, ^118^ Although none of these models fully replicates the complex pathophysiology of human AD, each offers distinct advantages, providing crucial insights that enhance our understanding of the disease and guide the search for effective therapeutic strategies.^108^ For example, AD animal models have shown that cognitive deficits do not depend solely on neuronal loss but also occur at an early stage when altered synaptic function and, consequently, abnormal network activity are pivotal aspects.^94^ This has raised the possibility of targeting AD before the damage becomes irreversible (i.e., when neuronal degeneration occurs) by restoring synaptic plasticity and network function, and, in this regard, NIBS might be critical.

Crucially, the same pathways influenced by NIBS techniques in rodents, such as the Fyn signaling one, also play a role in AD models. Indeed, Fyn is a non‐receptor tyrosine kinase involved in various processes, including synaptic function and plasticity.^119^ Fyn is activated by Aβ oligomers through their interaction with cellular prion protein and metabotropic glutamate receptors, leading to aberrant NMDA receptor hyperactivation and excitotoxicity.^120^, ^121^ Additionally, Fyn phosphorylates tau, promoting its pathological aggregation and mislocalization, which further exacerbates neuronal dysfunction.^122^ RTMS has been suggested to influence Fyn‐related pathways in animal models, potentially offering a neuroprotective approach in AD by restoring synaptic balance and preventing excitotoxic damage. In transgenic mice expressing human *APP* (*hAPP*), these pathways contribute to hAPP/Aβ‐induced neuronal and behavioral deficits relevant to cognitive decline. Elevated Fyn expression indeed exacerbates neuronal impairments, even under relatively moderate Aβ levels, while inhibiting Fyn activity has shown potential in mitigating amyloid‐induced dysfunctions.^123^, ^124^ These findings are pivotal as they suggest that NIBS may directly modulate the neurobiological pathways underlying AD neuropathology and its associated cognitive impairments.

Therefore, AD animal models have also been employed to investigate the neurobiological and therapeutic effects of different rTMS protocols. The following sections present an overview of the available evidence based on the specific mechanisms explored.

## 4 NEUROBIOLOGICAL EFFECTS OF rTMS IN AD ANIMAL MODELS

### 4.1 Effects on synaptic structural proteins

As mentioned, rTMS influences synaptic plasticity not only through functional changes but also through structural synaptic modifications. Indeed, a study examined SAMP8 mice, that is, a spontaneous model that overexpresses *APP* and exhibits all the neuropathological hallmarks of AD, including age‐associated hippocampal Aβ deposition, synaptic dysfunction, and impaired cognitive functions.^125^, ^126^ Compared to other transgenic AD models, SAMP8 better replicates the temporal sequence of critical neuropathological events, such as marked dendritic spine loss and late amyloid plaque deposition, making it a valuable tool for advancing therapeutic approaches.^127^ Notably, HF‐rTMS (5 Hz) induced upregulation of synapsin (SYN)/postsynaptic density protein 95 (PSD‐95), a marker of hippocampal synaptic plasticity.^125^ These changes correlated with cognitive improvements, suggesting a relevant link between this postsynaptic protein, hippocampal LTP, and cognitive performance. Similarly, a 25 Hz HF‐rTMS protocol in 3xTg AD mice reversed the reduction in PSD‐95 and SYP, respectively postsynaptic and presynaptic proteins, which are characteristically decreased in AD.^128^, ^129^ These reductions correlate with Aβ oligomer levels and dementia severity, highlighting the therapeutic potential of rTMS.^130^ Another study further explored the effects of rTMS treatments on PSD‐95 by comparing two different protocols—LF (1 Hz) and HF‐rTMS (10 Hz)—in an AD mouse model induced via intraperitoneal scopolamine injections.^131^ The focus was on micro ribonucleic acids (miRNAs), small non‐coding RNAs whose dysregulation has been implicated in the pathophysiology of numerous neurodegenerative diseases.^132^ Indeed, rTMS may exert its effects, at least in part, through modulation of the expression profile of multiple miRNAs.^133^, ^134^ Notably, both protocols effectively modulate the miRNA‐567/neurogenic differentiation 2 (NEUROD2, a transcriptional regulator involved in neuronal differentiation)/PSD‐95 axis, specifically by inhibiting the expression of miRNA‐567.^131^ This inhibition led to the upregulation of downstream effectors NEUROD2 and PSD‐95, which, as previously mentioned, are characteristically reduced in AD.^135^, ^136^ Importantly, NEUROD2 is critical for hippocampal mossy fiber synapses, and changes in the miRNA‐567/NEUROD2/PSD‐95 axis in mouse models were correlated with the development of AD symptoms.^131^, ^137^

These aspects highlight that rTMS protocols induce not only transient changes (e.g., in membrane channel kinetics) but also structural synaptic modifications, which could represent the neurobiological underpinnings of the long‐term changes induced by these techniques and, therefore, of the lasting therapeutic potential of NIBS protocols.

### 4.2 Effects on neurotransmitter circuits

The hypothesis of cholinergic circuit dysfunction as a key factor in the pathophysiology of AD is one of the most extensively studied theories.^138^ It underpins the use of therapies based on inhibitors of AChE, the enzyme responsible for degrading acetylcholine (ACh).^139^, ^140^ McNerney et al. investigated the effects of HF‐rTMS (10 Hz, applied daily for 2 weeks or twice weekly for 6 weeks) in 3xTgAD mice and demonstrated that, regardless of the protocol, rTMS increases ACh levels by modulating AChE activity.^141^ A recent study conducted on an AD model induced by intracerebroventricular injection of Aβ42 in mice compared the effects of two distinct rTMS protocols—specifically, HF‐rTMS (20 Hz) and LF‐rTMS (1 Hz)—in terms of neurotransmitter circuits and neurogenic signaling.^142^ While both protocols improved cognition‐related behaviors, the authors identified distinct neurobiological profiles. Indeed, both stimulation frequencies showed a marked enhancement in dopaminergic transmission, as highlighted by an increase in dopamine receptor 4 (DR4) expression within both the hippocampus and cerebral cortex. However, the most significant increase in DR4 was observed in the hippocampus following HF‐rTMS, aligning with experimental evidence demonstrating Aβ degradation and restoration of LTP‐like mechanisms after dopaminergic agonists administration.^143^, ^144^ Previous studies on animal models reinforced these findings by demonstrating that DR4 receptors play a significant role in working memory tasks and restoring hippocampal LTP in aged mice.^145^, ^146^ These data are consistent with results obtained in a rat model of Parkinson's disease (PD), in which HF‐rTMS (10 Hz) preserved the survival of dopaminergic neurons, reasonably by upregulation of neurotrophic/growth factors.^147^ Moreover, previous research documented the effect of rTMS treatments on serotonergic circuitry in rats.^148^ This is particularly significant given that depression, aside from being a risk factor that accelerates AD progression, is independently associated with hippocampal atrophy and memory deficits in both rats and humans.^149^, ^150^, ^151^ These findings suggest a potential common neurobiological substrate between AD and depression, and rTMS, at least in part by modulating serotonergic circuitry, identified as relevant in human AD pathophysiology, might improve both conditions by modulating shared neuronal elements.^152^, ^153^, ^154^

Notably, Aβ accumulation inhibits glutamate reuptake in the synaptic cleft, leading to overactivation of extracellular NMDA receptors.^155^ This results in an abnormal calcium influx and triggers the cascade of events associated with glutamatergic excitotoxicity, as already mentioned.^156^ These processes have been documented well before the appearance of amyloid plaques, making them detectable in the earliest stages of the disease.^157^, ^158^ Indeed, synaptic dysfunction and impaired glutamate homeostasis have been observed in the pre‐symptomatic stages of AD, before significant amyloid deposition occurs.^159^, ^160^ Consistently, current research suggests that soluble Aβ oligomers, rather than mature plaques, are responsible for disrupting synaptic plasticity and altering glutamatergic transmission early in the disease process.^158^, ^161^ Therefore, glutamatergic excitotoxicity may play a crucial role as a pathological mechanism driving synaptic dysfunction from the onset of the disease.^162^ A study in 3xTg‐AD mice demonstrated that HF‐rTMS (25 Hz), likely acting via the PI3K/Akt pathway, upregulates astrocytic glutamate transporter 1 expression.^128^, ^163^ This enhancement of glutamate reuptake helps to restore synaptic plasticity, reduce neuronal loss and cell apoptosis, and improve spatial learning and memory. Moreover, the same protocol increased the levels of NR2B, characteristically reduced in both 3xTg‐AD mice and AD patients.^164^ This suggests that rTMS yields the potential to modulate glutamatergic transmission at multiple levels and thus attenuate excitotoxic damage by normalizing different aspects of its function.

Interestingly, beyond the role of glutamatergic excitotoxicity in the pathophysiology of AD, recent studies have identified the involvement of the GABA circuit as well, which is the principal inhibitory neurotransmitter in the CNS.^165^, ^166^, ^167^ Wang et al. investigated the effects of HF‐rTMS (20 Hz) on 5xFAD transgenic mice, focusing on the GABA type A receptor subunit gamma2 (GABRG2)—a key subunit of the GABA receptor essential for inhibitory neurotransmission and synaptic plasticity, though its role in AD remains unclear—and the synaptosomal‐associated protein 25 (SNAP25), a vesicle fusion protein crucial for proper neurotransmitter release, neuronal excitability, and neurotransmitter balance.^168^, ^169^, ^170^, ^171^ Notably, SNAP25 has already been implicated in AD pathogenesis due to its involvement in neuroinflammation, Aβ deposition, and other neurodegenerative changes.^172^ Their findings suggest that rTMS may influence GABRG2, enhancing GABAergic neuron function and GABA expression. This, in turn, appears to affect SNAP25, which regulates the vesicle protein complex to protect synapses, promoting the survival of both glutamatergic and GABAergic neurons and fostering dendritogenesis.^171^, ^173^ Such a virtuous cycle may be relevant in alleviating AD symptoms.

This evidence suggests that rTMS protocols can modulate the activity of numerous neurotransmitter circuits that are characteristically disrupted in AD. Moreover, they appear capable of restoring the excitation/inhibition balance by targeting both excitatory (glutamatergic) and inhibitory (GABAergic) pathways, a pivotal aspect from a therapeutic perspective.

### 4.3 Effects on neurotrophic factors

Among the processes involved in AD pathophysiology, a significant age‐related decrease in neurotrophic factors such as nerve growth factor (NGF) and BDNF has also been observed.^174^ Significant evidence for these mechanisms comes from studies using an Aβ1‐42–induced toxicity model in rats.^71^ Specifically, rats received bilateral injections of Aβ1‐42 oligomers into the dentate gyrus, which exert neurotoxic effects by suppressing neurotrophic factors such as BDNF and NGF and inducing early synaptic dysfunction. This process promotes amyloid plaque formation and leads to persistent memory deficits.^175^, ^176^, ^177^, ^178^ Notably, injections of exogenous neurotrophins into the hippocampus have been shown to reverse spatial memory deficits in animal models of TBI.^179^ However, since neurotrophins cannot cross the blood–brain barrier (BBB), there is considerable interest in strategies to induce their expression through noninvasive methods.^71^ RTMS has shown promise in this regard, as it increases the expression of endogenous neurotrophins in the CNS of healthy rats.^61^, ^69^ Furthermore, AD animal models display a significant reduction in the expression of NMDA receptor subunits (including NR1, NR2B, and NR2A), and neurotrophins like BDNF and NGF upregulate NMDA receptor expression in the hippocampus of rats.^180^, ^181^, ^182^ Consequently, Tan et al. examined the effects of LF‐rTMS (1 Hz) in this AD model, demonstrating that it promotes hippocampal expression of endogenous neurotrophins and NMDA receptors, which in turn reduces the spatial memory deficits caused by Aβ1‐42 injection.^71^ However, only the amyloid‐injected rats showed an increase in LTP—a finding the authors attributed to a “ceiling effect,” which explains the modulation of LTP solely in animals with specific impairments in these mechanisms.^183^ These findings align with other research showing that LF‐rTMS increases neurotrophin levels in both healthy rats and models of vascular dementia, likely enhancing release from various CNS cell types.^184^, ^185^, ^186^, ^187^ As suggested, the effects on LTP may depend directly on increased neurotrophin levels or their ability to upregulate NMDA receptor expression.^69^, ^71^, ^185^ Indeed, the extent to which LTP relies on neurotrophin‐dependent mechanisms remains unclear. Numerous studies suggest that higher neurotrophin levels—especially BDNF—enhance synaptic plasticity and LTP.^188^ However, other research indicates that LTP can be modulated, at least partially, independently of direct neurotrophin signaling.^189^, ^190^, ^191^ The modulation of LTP becomes even more complex in pathological conditions, leading to the reasonable hypothesis that NIBS protocols may operate through multiple non‐mutually exclusive mechanisms.^192^, ^193^ Thus, rTMS might compensate for synaptic deficits by increasing neurotrophin release while also activating additional pathways, such as NMDA receptor modulation or changes in intrinsic excitability, in response to the specific alterations due to the pathological condition. Further studies are needed to clarify the specific mechanisms of action of rTMS.

Notably, rTMS could enhance BDNF and NGF expression irrespective of frequency, as both LF and HF‐rTMS improved their reduced levels in a mouse AD model based on scopolamine injection.^131^ However, a previously discussed study observed distinct changes in neurotrophic factors between HF and LF‐rTMS.^142^ Indeed, only HF‐rTMS demonstrated increases in the expression of BDNF, nestin, and NeuN (neuron‐specific nuclear protein, Fox‐3, Rbfox3, or hexaribonucleotide‐binding protein‐3) in the cerebral cortex, as well as BDNF in the hippocampus. Notably, while BDNF, belonging to the TrkB pathway, is a key neurotrophic factor regulating neurogenesis, differentiation, and neuronal survival—its increase correlating with spatial working memory through NMDA receptor mediation—nestin and NeuN indicate heightened neurogenic activity.^194^, ^195^, ^196^ Thus, HF‐rTMS seems to exert more pronounced effects on the pathophysiological mechanisms underlying AD.^142^ Consistently, in *APP/PSEN1* double‐mutant transgenic mice, an HF‐rTMS treatment (5 Hz) highlighted an improvement in learning, memory, and cognitive impairment also due to a specific modulation on the BDNF‐TrkB signaling pathway.^197^ Similarly, another study showed that HF‐rTMS enhances *BDNF* gene expression, which correlates with AChE activity.^141^ Both are crucial in the pathophysiology of AD, aligning with previous research that has identified a correlation between cortical AChE and BDNF levels.^198^

Therefore, rTMS protocols may increase the expression of neurotrophic factors that can, directly and indirectly, counteract many of the characteristic alterations of AD pathophysiology, including LTP disruption, cholinergic deficit, and NMDA receptor expression.

### 4.4 Effects on ionophoric channels

It is well established that Aβ increases cortical excitability by modulating the molecular expression of various ion channel types.^199^ Since these processes appear to play a pivotal role in AD pathophysiology, several studies have investigated the therapeutic potential of rTMS in altering ion channel expression. Consistently, Wang et al. focused on 3xTg AD model mice, suitable for examining the effects of intracellular soluble Aβ accumulation, which precedes the formation of characteristic extracellular plaques and is now considered an early event in AD pathophysiology.^200^, ^201^, ^202^ Using different frequencies (ranging from 1 to 15 Hz), the authors demonstrated that rTMS improves performance in a spatial learning task, likely by reducing Aβ accumulation and counteracting the suppressive effects of soluble Aβ1‐42 on a class of ionic channels, namely the large conductance calcium‐activated potassium (Big‐K [BK]) ones, whose activation was associated with enhanced LTP.^202^ Hypothetically, the effects of rTMS arise from the modulation of IEGs, particularly *Homer1a*.^202^, ^203^ Thus, rTMS has the potential to counteract the neuropathological process from its earliest stages, thereby improving cognitive performance. Indeed, rTMS could disrupt a vicious cycle wherein Aβ1‐42‐induced suppression of BK channels (associated with increased excitotoxicity due to excessive glutamate release from presynaptic terminals) enhances neuronal excitability, which in turn promotes Aβ1‐42 production.^204^, ^205^, ^206^, ^207^ Notably, this mechanism may also underlie the pathological increase in cortical excitability observed in both AD animal models and patients, which fosters epileptiform activity (even subclinical) that exacerbates and accelerates cognitive decline.^208^, ^209^, ^210^, ^211^ Indeed, neuronal hyperactivity is detectable both in the cortex and hippocampus of AD models and could stem from intrinsic hyperexcitability or reduced inhibition, which might be responsible for the increased threshold required for LTP induction, while LTD remains facilitated.^212^, ^213^, ^214^, ^215^, ^216^, ^217^ By stabilizing neuronal excitability in both animal models of AD and humans, rTMS could help restore the physiological mechanisms of LTP/LTD.^20^, ^218^ This may also involve increased expression of genes critically involved in synaptic plasticity, such as *c‐Fos* and *Zif268*, acting as third messengers regulating BDNF gene expression in both cortical and hippocampal regions.^45^ Interestingly, in a mouse model of depression, a similar pattern of hyperexcitability has been observed in brain regions implicated in the disease's pathophysiology.^149^, ^219^ Notably, HF‐rTMS (10 Hz) has been shown to normalize cortical hyperexcitability, similar to studies previously discussed, through the modulation of *Homer1a*, which activates group I metabotropic glutamate receptors (mGluRs) from inside the neuron, leading to crucial facilitation of BK channels. Indeed, BK channel suppression leads to neuronal death because of a perturbation of Ca^2+^ homeostasis and, thus, an excessive glutamate release.^220^, ^221^

These findings suggest that rTMS targets a neurobiological mechanism disrupted across multiple pathological conditions and might represent an essential tool to restore physiological neuronal membrane potential, a key aspect to counteract the pathological neuronal hyperexcitability typical of AD pathophysiology.

### 4.5 Effects on production/clearance of neurotoxic “amyloid cascade” products

NIBS protocols might also directly modulate the pathological cascade of toxic product accumulation, counteracting this critical process to prevent further damage. For example, an HF‐rTMS (25 Hz) protocol in 3xTg AD mice reduced hippocampal Aβ1‐42 levels, likely through PI3K/Akt pathway activation via the modulation of BACE1‐mediated *APP* cleavage.^128^, ^222^ In addition, LF‐rTMS (1 Hz) in the *APP23/PSEN45* double transgenic mouse model of AD can counteract neuropathological damage, thereby improving hippocampal LTP as well as spatial memory and learning, both of which are impaired in these animals.^218^ Reasonably, LF‐rTMS reduced BACE1 expression (elevated in both animal models and AD patients), leading to decreased production of APP and its C‐terminal fragment products.^223^ This suggests that this NIBS protocol also has the potential to directly intervene in disease pathophysiology by disrupting the amyloid cascade and preventing subsequent damage. However, since a reduction in neuritic plaques was also observed, the authors hypothesize that the treatment may have also enhanced Aβ clearance, implying that the therapeutic benefit may be twofold: reducing synthesis and increasing elimination of neurotoxic products.^218^

Consistently, in 5xFAD mice, HF‐rTMS (20 Hz) can mitigate long‐term memory deficits by enhancing the drainage efficiency of the brain parenchyma (and reducing neuroinflammatory mechanisms, as discussed in the following section).^224^ Specifically, interstitial Aβ reaches the meningeal lymphatics via the glymphatic system and is subsequently transported to the deep cervical lymph nodes. Notably, rTMS appears to facilitate its clearance through this pathway.^225^, ^226^, ^227^ These findings underscore the role of impaired clearance of neurotoxic products in AD pathophysiology, as aging‐related dysfunction of the glymphatic system or meningeal lymphatics promotes Aβ and Tau accumulation in both animal models and humans.^228^, ^229^ Thus, rTMS holds the potential to restore the proper functioning of systems responsible for eliminating toxic products, thereby preventing their buildup. Moreover, another system that eliminates toxic metabolites relies on autophagic mechanisms, facilitating the controlled reduction of aged or waste cellular products.^230^ Indeed, the Apolipoprotein E (*ApoE*) gene signaling mediates lysosomal degradation and the clearance of Aβ and Tau, and it has been demonstrated that HF‐rTMS can enhance their autophagic removal through the downregulation of *ApoE* expression in *APP/PSEN1* mice.^197^ The role of *ApoE*, particularly the ε4 subtype, in increasing the risk of AD is well established, likely due to its influence on these processes, which might explain the early brain network changes observed between carriers and non‐carriers.^231^, ^232^ Therefore, this NIBS protocol might represent a promising approach for modulating this complex pathway and reducing the risk associated with impaired clearance of toxic metabolites.

Notably, rTMS appears to influence not only Aβ‐related production pathways but also those involving Tau. In an AD mouse model induced by Aβ1‐42 injection, both LF‐rTMS (1 Hz) and HF‐rTMS (10 Hz) protocols enhanced cognitive performance through the activation of β‐catenin via the regulation of glycogen synthase kinase‐3β (GSK‐3β) and Tau.^233^ Specifically, GSK‐3β phosphorylates both Tau and β‐catenin, and the proper phosphorylation of the former ensures the correct functioning of the latter, which supports neuronal survival.^234^ Interestingly, *PSEN1*, which is also modulated by rTMS, influences GSK‐3β activity, further highlighting its critical role in this cascade of events.^235^ Although the data are not entirely consistent—some studies have shown that pTau inhibits β‐catenin levels, while others have reported opposite findings, likely due to differences in study models and disease stages—the ability to modulate GSK‐3β activity emerges as a critical factor underlying the therapeutic effects of rTMS.^233^, ^234^ This is particularly compelling considering recent evidence suggesting that GSK‐3β could represent a potential link between diabetes mellitus (DM) and AD (sometimes referred to as type 3 diabetes).^236^ Consistently, GSK‐3β is involved in both Tau phosphorylation and the insulin/PI3K/Akt signaling pathway. Disruptions in this latter signaling pathway, which regulates glucose metabolism in the brain, can lead to the formation of pTau in both AD animal models and human patients.^237^ At the same time, insulin resistance may contribute to Aβ deposition.^238^ Finally, a study in the SAMP8 mouse model showed that an antisense oligonucleotide against GSK‐3β improved learning and memory and increased Nuclear factor erythroid 2‐related factor 2 levels, reducing oxidative stress and Tau phosphorylation.^239^

These insights might explain promising findings showing that medications used to treat DM and implicated in these signaling pathways have beneficial effects in improving cognitive performance in AD models and patients.^236^, ^240^, ^241^, ^242^ This link is further supported by growing evidence that DM and AD not only share physiopathological mechanisms but also exhibit similarities in clinical and neuroimaging profiles. Indeed, an association between olfactory dysfunction and specific memory impairments in patients with prediabetes and diabetes has been highlighted.^243^ This led to the intriguing hypothesis that olfactory dysfunction could predict memory decline (i.e., one of the earliest manifestations across the AD spectrum) in these populations. Furthermore, the same research group identified marked neurodegeneration in the right insula and inferior temporal gyrus, specifically linked to cognitive impairment in patients with advanced diabetic retinopathy.^244^, ^245^ Hence, these conditions may fall within a neuropathological continuum, and their coexistence could accelerate cognitive decline. Importantly, both DM and AD are marked by early impairments across multiple sensory modalities, ultimately leading to memory deterioration.^246^, ^247^ This aligns with the hypothesis suggesting a disruption in the internal models of sensorimotor interactions with the external environment.^54^, ^248^ Such a disruption could plausibly trigger a vicious cycle, further exacerbating ongoing damage from metabolic alterations and network reorganization meant to compensate for deficits.^249^, ^250^

Importantly, NIBS protocols have been shown to enhance peripheral metabolism, including glucose regulation. For instance, a recent study utilizing a rat model of type 2 DM implemented three sessions of iTBS.^251^ After a 10‐day treatment, significant improvements were noted in body weight, fasting plasma glucose, glucose tolerance, and insulin sensitivity. These benefits persisted after 21 days, with continued weight loss and improved insulin resistance, alongside decreased circulating levels of lipids. Importantly, RNA sequencing analyses further revealed the specific modulation of genes associated with diabetes, obesity, fatty acid synthesis, and appetite in both the liver and hypothalamus, indicating a complex signaling mechanism that links central and peripheral metabolic regulation via rTMS—an area that warrants further investigation.

These findings underscore the potential of rTMS to modulate peripheral metabolic processes critical to AD pathophysiology, reinforcing the notion that its therapeutic effects arise from multiple complementary molecular mechanisms.

### 4.6 Effects on neuroinflammation and oxidative stress

In the CNS, neuroinflammatory responses are marked by the activation of microglial cells and astrocytes, which trigger a cascade of pro‐inflammatory mediators alongside the generation of reactive oxygen species (ROS). These elements collectively sustain the neuroinflammatory process, a phenomenon known as reactive gliosis, which, in turn, disrupts synaptic connectivity, alters neurotransmitter homeostasis, and induces glutamate excitotoxicity, leading to maladaptive changes to synaptic plasticity.^252^, ^253^ Consistently, microglial cells, that is, macrophages resident in the CNS, have a well‐established role in the pathophysiology of neurodegenerative diseases.^254^ Notably, beyond its effect on Aβ clearance, HF‐rTMS can counteract glial activation in the brains of 5xFAD mice, leading to cognitive improvements.^224^ Considering that neuroinflammatory processes, particularly in the chronic phase, cause severe neuronal and BBB disruption and that overactivation of microglia and astroglia by Aβ oligomers or plaques appears to be an early event in AD pathophysiology, interventions targeting these processes could be critical in combating neuropathological damage.^109^, ^255^, ^256^ HF‐rTMS may not only modulate glial cells directly but also reduce their hyperactivity by promoting the clearance of toxic products or regulating the release of neuromodulators involved in controlling their activity.^224^, ^257^, ^258^ Furthermore, HF‐rTMS (25 Hz) in 3xTg AD mice can mitigate the abnormally high oxidative stress characteristic of this disease model by reducing the production of pro‐oxidative molecules such as ROS and malondialdehyde while increasing the levels of antioxidants like superoxide dismutase and glutathione.^128^ Indeed, rTMS appears to reduce microglial activation in the dentate gyrus of the hippocampus and decrease the release of pro‐inflammatory cytokines.^128^ Similar results were observed in a study involving 5xFAD mice, which exhibited significantly elevated Aβ levels along with early signs of cognitive impairment, as demonstrated by worsened performance in behavioral tasks compared to wild‐type controls.^259^ This early accumulation of Aβ disrupts synaptic plasticity and triggers microglial activation via pro‐inflammatory cytokines. HF‐rTMS (20 Hz) reduced neuroinflammation and hippocampal Aβ burden while improving synaptic plasticity and rescuing early cognitive deficits in this model.^259^ The expression of pro‐inflammatory factors was particularly diminished in the hippocampus, suggesting that the therapeutic effects of rTMS may be partly attributed to its ability to attenuate neuroinflammatory processes.^259^, ^260^, ^261^ This is likely achieved by inhibiting the activity of the PI3K/Akt/nuclear factor kappa B signaling pathway, which is associated with the early expression of pro‐inflammatory cytokines.^259^, ^262^, ^263^ A study consistently evaluated the effects on neuroinflammation of an iTBS protocol using a trimethyltin‐induced AD‐like model.^264^ After 3 weeks, iTBS reduced inflammation and increased anti‐inflammatory molecules, specifically linked to reversing the downregulation of phosphorylated forms of Akt and the mammalian target of rapamycin. Moreover, iTBS produced beneficial effects on cognition, anxiety‐related, and aggressive behavior.

Therefore, rTMS could be a key tool to counteract the earliest pathogenetic mechanisms of AD, which can be traced back to neuroinflammation, thus preventing the initiation of the cascade leading to the accumulation of toxic products.

Table 1 presents an overview of the evidence discussed by animal models regarding the neurobiological mechanisms that underlie the efficacy of rTMS treatments, organized into healthy animal models (first section) and disease models (second section).

**TABLE 1:** Overview of evidence from animal models regarding the neurobiological mechanisms that support the efficacy of rTMS treatments, categorized into healthy animal models (first section) and disease models (second section).

Reference	Animal model type	Features of the NIBS protocol	Main findings
**Healthy animal models**			
^148^	Adult (3‐ to 4‐month‐old) male Long–Evans rats	rTMS: 25 Hz, 2 s, once daily for 7 days	LTP enhancement in the perforant path synapse in the dentate gyrus. Modulation of serotoninergic circuitry, as rTMS suppressed the serotonin‐dependent, potentiating action of fenfluramine on population spike in the dentate gyrus
^36^	Male Wistar rats at 7 weeks old	rTMS: 20 Hz, 2 s, 6 sessions	Enhanced expression of *Fos* in frontal cortex, striatum, cingulate cortex, thalamus, and occipital cortex (not in other regions)
^37^	Male Sprague–Dawley rats	rTMS: 15 Hz, 3 s, 1 session or once daily for 3 days	Alteration of the hypothalamic‐pituitary‐adrenal stress axis with a specific time‐course, with lower corticosterone concentrations at 6 and 24 h following a single application
^39^	Male C57Black mice	rTMS: 20 Hz, 2 s, 20 times/day, 20, 30 or 40 days or acutely for 1 day	Increased expression of genes encoding neurotransmitter transporters and endoplasmic reticulum‐stress proteins. Expression changes in many of these genes also 10 days after the last rTMS treatment
^40^ ^(p20)^	Young adult male Wistar rats (3 months)	rTMS: 20 Hz, 2.5 s, single session	Continuous reduction in arginine vasopressin release (up to 50%) and selective stimulation of the release of taurine, aspartate and serine within the hypothalamic paraventricular nucleus. Elevation of dopamine in the dorsal hippocampus
^43^	Female Wistar rats	rTMS: 20 Hz, 3 s, single session	Increased density of 5‐HT_1A_‐binding sites in frontal cortex, cingulate cortex, and anterior olfactory nucleus and of NMDA binding sites in ventromedial hypothalamus, basolateral amygdala, and parietal cortex
^45^	Adult male Dark Agouti rats (Charles River)	Single 2‐s episodes of iTBS, 10 Hz or 1 Hz rTMS	c‐*Fos*: 1 and 10 Hz rTMS enhanced its expression in all cortical areas, while iTBS only in limbic cortices. Zif268: iTBS increased its expression in almost all cortical areas, while 10 Hz rTMS only in the primary motor and sensory cortices. 1 Hz rTMS had no effect on cortical zif268 expression
^48^	Adult male Dark Agouti rats (Charles River)	rTMS: 1 Hz, three 20‐min trains applied at 25‐min intervals. iTBS and cTBS: five blocks separated by 15 min	Acutely (2 h), all protocols decreased GAD67 in frontal, motor, somatosensory, and visual cortex while increasing GAD65 and GAT‐1 to varying degrees (iTBS with the strongest effect). Initial reduction in GAD67 reversed after 1 day, leading to a sustained increase (up to 7 days), particularly in frontal cortex following iTBS and cTBS, and across all areas with 1 Hz rTMS. While GAD65 and GAT‐1 normalized after 1 day with iTBS and cTBS, 1 Hz rTMS induced a continuous increase in both during the 7 days
^49^	Adult male Sprague–Dawley rats	rTMS: 20 Hz, 10 s, once per day, 14 days	Induction of c‐fos mRNA and protein in the parietal cortex (layers I–IV and VI) and in few scattered hippocampal neurons
^51^	Male adult (3 months old) or young (from 4 weeks old) for the long‐term paradigm Wistar rats	rTMS: 20 Hz, 2.5 s, 1000 stimuli in a single session or 150 stimuli/day in 5‐day series separated by 2‐day pauses for 11 weeks	No evidence for structural brain damage and cognitive impairment: no increase in GFAP, nor cognitive deficits in spatial learning and memory (as assessed in the Morris water‐maze task) or in olfactory‐cued short‐term memory performance (as assessed in the social discrimination procedure). Increased activation of the alpha‐secretory non‐amyloidogenic APP pathway and release of sAPP
^52^	Male Sprague–Dawley rats	rTMS: 0.5 Hz, 1000 s, once daily, 15 consecutive days	Increased glutamate and GABA concentrations in the hippocampus and striatum, while both decreased in the hypothalamus. No changes in glutamate and GABA content in the midbrain
^56^	Male C57Black R6 mice (8 weeks old)	rTMS: 20 Hz, 2 s, 20 times/day, acutely for 1 day or chronically for 20 days	Acute rTMS increased c‐fos expression, whereas chronic rTMS led to its downregulation. Chronic rTMS decreased serotonin transporter mRNA levels, leading to reduced serotonin uptake and binding, while DAT and NET transporter mRNA levels increased, with NET showing corresponding changes in uptake and binding, while DAT did not
^44^	Male Wistar rats (10–13 weeks old)	rTMS: 25 Hz, 1 s, single session	Increase in extracellular dopamine concentrations in the dorsolateral striatum continuously, lasting for at least 140 min
^58^	Male Wistar rats (from 4 weeks old)	rTMS: 20 Hz, 2.5 s, 150 stimuli/day, 5‐day separated by 2‐day intervals for 11 weeks	Increase in BDNF mRNA in the hippocampal areas CA3 and CA3c, the granule cell layer, and in the parietal and piriform cortex. Significant increase in CCK mRNA in all brain regions examined
^61^	Male Sprague Dawley rats (60 days old)	rTMS: 1 Hz, 900 s, applied continuously or 9 trains of 100 pulses at 20 Hz (5 s/train). Daily sessions, 10 days (2‐week period)	Lasting effects of 20 Hz (but not 1 Hz) rTMS on neuroplasticity, specifically BDNF, GluR1, or pGluR1 levels in all examined regions. Alterations in these markers remained 3 days after the last rTMS session
^68^	129/SvEv male mice (10–12 weeks old)	rTMS: 15 Hz, 5 s, 5‐days, progressive number of trains (one treatment delivered on day 1, up to five on day 5)	Increased total spine density in apical and basal dendrites of pyramidal neurons in layer II/III of the primary motor cortex, with a predominance of thin spines and increased dendritic complexity at both apical and basal dendrites
^69^	Male Sprague Dawley rats (10‐week‐old)	rTMS: 5 Hz, 2.5 min, 1600 stimuli, five sessions daily for 5 consecutive days	Improved BDNF–TrkB signaling by increasing the affinity of BDNF for TrkB and heightened downstream ERK2 and PI‐3K activities in prefrontal cortex and in lymphocytes. The elevated BDNF–TrkB signaling is accompanied by an increased association between the activated TrkB and NMDA receptor
^71^	Male Sprague Dawley rats (2.5‐month‐old)	rTMS: 20 burst trains, each consisting of 20 pulses at 1 Hz (in total 400 stimuli), one session for 14 consecutive days	Enhancement of hippocampal endogenous neurotrophins (i.e., NGF, BDNF) and NMDA receptor expressions
^75^	Male Wistar rats (4 weeks old)	rTMS: 25 Hz, ten 1‐s trains, 4 times per day (1000 pulses/day) for 7 days	LTP enhancement at the level of CA1 pyramidal cells
^88^	Adult male Wistar rats	rTMS: 5 Hz, 1 session daily (20 burst trains/session, 20 pulses/train, in total 400 stimuli) for 14 consecutive days	Enhancement in spatial learning/memory and reversal learning/memory, as well as in LTP, depotentiation, paired‐pulse facilitation, expressions of BDNF, presynaptic protein SYP, and postsynaptic protein NR2B
**Disease animal models**			
^125^	Male SAMP8 mice (7‐month‐old)	rTMS: 5 Hz, 4 min and 38 s (session duration), 2 sessions (1000 pulses in 10 trains) for 14 consecutive days	Amelioration of the learning and memory dysfunction (as assessed thorough the Morris water‐maze task) associated to an upregulation of hippocampal SYN/PSD95 proteins
^128^	Homozygous 3xTg‐AD mice (6–8 months old)	rTMS: 25 Hz, 10 trains of 100 pulses (4 s each train), 1000 stimuli/day for 28 days	rTMS improved cognitive function and glucose metabolism, reduced hippocampal Aβ1‐42 levels, and ameliorated oxidative stress by reducing the production of pro‐oxidative molecules while increasing the levels of antioxidants. Moreover, it alleviated neuroinflammatory response, enhanced synaptic plasticity, and reduced neuronal loss and cell apoptosis, accompanied by activation of PI3K/Akt/GLT‐1 pathway. Finally, it reversed the downregulation of NR2B levels commonly observed in AD
^131^	C57BL/6 mice (8‐week‐old) used as AD‐related MCI model throrugh an intraperitoneal injection of scopolamine	rTMS: 1 and 10 Hz, 2 sessions (consisting of 1000 pulses in 10 trains) separated by an interval of 2 min	rTMS enhanced cognitive function and mitigated brain tissue damage (linked to the restoration of BDNF and NGF production). Moreover, it downregulated miR‐567 while upregulating NEUROD2 and PSD95 expression. When miR‐567 levels were artificially increased, the beneficial effects of rTMS were counteracted. These alterations were associated with the downregulation of NEUROD2 and PSD95
^133^	Male Sprague–Dawley rats (7‐week‐old) used as model of focal cerebral ischemia	rTMS: 10 Hz, 3 s, repeated 10 times (300 pulses/day), daily sessions for a 7‐day period	rTMS promoted adult neural stem cells proliferation in the subventricular zone after focal cerebral ischemia and this protective effect was associated with the miR‐25/p57‐signaling pathway
^141^	3xTgAD female mice (12‐month‐old)	rTMS: 10 Hz, 10 min, every day for 2 weeks, or twice a week for 6 weeks.	Both 14 days and 6 weeks regimens increased acetylcholinesterase activity and BDNF gene expression, with a larger increase in the 6‐week group
^142^	C57BL/6 male mice (8 weeks old) as AD model induced by Aβ42 injection	rTMS: 20 Hz, 40 trains of 2 s duration, or continuous 1‐Hz, 1600 pulses/session, 2 weeks, 5 consecutive days in a week	Both the frequencies improved behavioural performance in Y‐maze test and novel object recognition task (tendency for further effects of high frequency in Y‐maze test), increased dopamine levels, and upregulated dopamine‐receptor 4, with higher response by high frequency stimulation. Only high frequency induced an elevation of BDNF levels and enhanced the expression of Nestin and NeuN in the brain tissue
^171^	C57Bl/6 mice (4‐month‐old) used for AD modeling as 5xFAD transgenic specimen	rTMS: 20 Hz, 20 bursts (each lasting 2 s), daily session for 14 days (after 14 days, a 7‐day recovery period was allowed)	Improvements in behavioral performance (as evaluated by social interaction changes, the open‐field test, the Y‐maze alternation test, and the Morris water maze test), enhanced functionality of GABAergic neurons, and reductions in Aβ deposition and neuroinflammation. Furthermore, evidence for a role of SNAP25 downstream of GABRG2 in mediating rTMS's effect
^197^	APP/PS1 double‐mutant transgenic mice (3‐month‐old)	rTMS: 5 Hz, 600 pulses (20 burst trains and 30 pulses each train), daily for 14 consecutive days.	rTMS alleviated cognitive impairments and AD‐like pathology, reducing pTau, APP, Aβ, and PP2A expression. Enhancement of BDNF‐TrkB signaling and increase in hippocampal autophagic activity, as indicated by a higher LC3II/LC3I ratio and a significant reduction in ApoE and p62
^202^	3xTgAD male mice (4‐ to 5‐month‐old)	rTMS: 1, 10 or 15 Hz, 5 s, once a day for 4 weeks	rTMS improved spatial learning (as assessed by the Morris water maze test) by counteracting the suppressive effects of soluble Aβ1‐42 on BK channels while also reducing Aβ levels. BK channel activation restores synaptic plasticity. Although the mechanism is unclear, rTMS‐induced Homer1a expression may mediate this effect
^218^	APP23/PS45 double mutant transgenic mice (1.5‐month‐old)	rTMS: 1 Hz, 20 burst trains (30 pulses/train), in total 600 stimuli, one session daily for 14 consecutive days	rTMS reversed the impairment of spatial learning and memory (as assessed by the Morris water maze test) and hippocampal CA1 LTP. Significant reduction of amyloid‐β precursor protein, its C‐terminal fragments (C99 and C89), and β‐site APP‐cleaving enzyme 1 in the hippocampus
^71^	Male Sprague Dawley rats (2.5‐month‐old) injected with incubated Aβ1‐42	rTMS: 1 Hz, 20 burst trains each consisting of 20 pulses at (in total 400 stimuli), 1 session for 14 consecutive days	The rTMS‐induced increment of neurotrophins (i.e., NGF, BDNF) up‐regulated hippocampal NMDA‐receptor expression, and rescued deficits in LTP and spatial memory
^224^	Female and male 5xFAD mice (4‐ to 5‐month‐old)	rTMS: 20 Hz, 100 sessions/day (40 burst trains/session), 14 consecutive days	rTMS increased the drainage efficiency of the glymphatic system in brain parenchyma and the meningeal lymphatics, with significant reduction of Aβ deposits, suppression of microglia and astrocyte activation, and prevention of decline of neuronal activity, as indicated by the elevated c‐*Fos* expression, in the prefrontal cortex and hippocampus
^233^	C57BL/6 mice (8 weeks old) as AD model induced by A*β*_1‐42_ injection	rTMS: 1 Hz or 10 Hz, 2 sessions (1000 pulses in 10 trains), 14 consecutive days	Both frequencies improved the cognitive function (as assessed thorough the Morris water‐maze task), suppressed neuron apoptosis, and increased the brain BDNF, NGF, and doublecortin levels. The injection of Aβ1‐42 also increased the expressions of p‐GSK‐3β, p‐Tau, and p‐β‐catenin and suppressed the total β‐catenin. After rTMS, the level of β‐catenin was restored, indicating the activation of β‐catenin signaling.
^259^	Adult male and female double transgenic 5xFAD (2‐month‐old)	rTMS: 20 Hz, 20 trains (20 pulses each), 14 consecutive days	rTMS reduced hippocampal Aβ and pro‐inflammatory cytokines (including IL‐6 and TNF‐α) levels, decreased activation of microglia, and regulated PI3K/Akt/NF‐κB signaling pathway
^264^	Male Wistar rats (2‐month‐old) as trimethyltin‐induced Alzheimer's‐like disease model	iTBS, 2 times per day for 15 days	iTBS decreased pro‐inflammatory cytokines and p‐Akt/t‐Akt, and anti‐inflammatory cytokines. Furthermore, it reverted the significant downregulation of phosphorylated forms of Akt and mTOR and improved cognition, as well as reduced anxiety‐related and aggressive behavior.
^219^	C57BL/6 male mice (15–20 weeks old) (model of depression)	rTMS: 10 Hz, 5 s, once a day for 4 weeks	rTMS significantly alleviated depression‐like behaviors, restoring both normal neuronal excitability and BK channel activity. Additionally, it reversed the reduced expression of the scaffold protein Homer1a in the cingulate cortex.
^186^	Male Sprague–Dawley rats as model of VaD through permanent middle cerebral artery occlusion	rTMS: 0.5 Hz, 2 times/day, and 30 pulses each time, different treatment duration (1, 7, 14, 21, and 28 day(s) after middle cerebral artery occlusion)	rTMS enhanced neurological severity score recovery on days 7, 14, 21, and 28. c‐Fos and BDNF expression was detected in the peri‐infarct cortex, with significantly higher levels at multiple time points
^185^	Male Wistar rats as model of VaD through bilateral carotid common artery ligation	rTMS: 0.5 or 5 Hz, 20 strings/day (10 pulses/string), 5 days in a row as a course, with a total of 6 courses and an interval of 2 days for each course	Both the rTMS protocols improved the learning and memory abilities (as assessed by the Morris water maze test), as well as the mRNA and protein expressions of BDNF, NMDAR1, and SYN. The ultra‐structures of synapses in hippocampal CA1 area in rTMS groups were reformed
^147^	Adult male Sprague–Dawley rats unilaterally injected with 6‐OHDA into the right striatum (model of PD)	rTMS: 10 Hz, 1 s, 20 min/day, treatments performed daily for 4 weeks	rTMS reduced amphetamine‐induced rotations, improved treadmill locomotion, and increased the number of tyrosine hydroxylase‐positive dopaminergic neurons and fibers in both the ipsilateral SNc and striatum. Elevation of the expression of BDNF, GDNF, PDGF, and VEGF, in the 6‐OHDA‐injected hemisphere and SNc
^50^	Male Sprague–Dawley rats (model of depression)	rTMS: 25 Hz, 2 s, single session	Strong increase of c‐*fos* expression predominately in the paraventricular nucleus and specific cortical regions, moderate in regions controlling circadian rhythms
^42^	Male Sprague–Dawley rats (model of depression)	rTMS: 25 Hz, 2 s, daily sessions, 7 or 10 days	Enhancement of apormorphine‐induced stereotypy and reduction of immobility in the Porsolt swim test
^251^	Male Wistar rats (4 weeks old) as model of T2DM through a high‐fat diet followed by hyperglycemia induction by intraperitoneal injections of streptoxolocin	iTBS, 3 sessions (each involving daily stimulation for 5 consecutive days followed by 2 non‐stimulation days)	After 10‐day, improvements in body weight, fasting plasma glucose, glucose tolerance, and insulin sensitivity. These benefits persisted after 21 days, with sustained weight loss and improved insulin resistance, alongside decreased circulating levels of cholesteryl esters, triglycerides, and ceramides. RNA‐sequence analyses showed a modulation of genes related to diabetes, obesity, fatty acid synthesis, and appetite in both the liver and hypothalamus

Abbreviations: (p)GluR1, (phosphorylated) glutamate receptor 1; 5‐HT_1A_, serotonin 1A receptor; 6‐OHDA, 6‐hydroxydopamine; AD, Alzheimer's disease; AKT, protein kinase B; *ApoE*, apolipoprotein E; *APP*, amyloid precursor protein; Aβ, amyloid‐beta; BACE, β‐site APP cleaving enzyme; BDNF, brain‐derived neurotrophic factor; BK, large conductance calcium‐activated potassium channels; CA, cornu ammonis; CCK, cholecystokinin; cTBS, continuous theta burst stimulation; CTF, C‐terminal fragment;DAT, dopamine transporter; DR4, dopamine receptor 4; ERK 1/2, extracellular signal‐regulated kinases ½; GABA, gamma‐aminobutyric acid; GABRG2, GABA type A receptor subunit gamma2; GAD65/67, glutamic acid decarboxylase 65/67; GAT‐1, GABA transporter 1; GDNF, glial cell line‐derived neurotrophic factor; GFAP, glial fibrillary acidic protein; GLT‐1, glutamate transporter 1; GSK‐3β, glycogen synthase kinase‐3β; iTBS, intermittent theta burst stimulation; LTP, long‐term potentiation; MCI, mild cognitive impairment; miRNAs, micro ribonucleic acids; mRNA, messenger ribonucleic acid; mTOR, mammalian target of Rapamicin; NET, norepinephrine transporter; NeuN, neuron‐specific nuclear protein; NEUROD2, neurogenic differentiation 2; NFG: nerve growth factor; NF‐κB, nuclear factor kappa B; NIBS, noninvasive brain stimulation; NMDA, N‐methyl‐D‐aspartate; NR2B, N‐methyl D‐aspartate receptor subtype 2B; PD, Parkinson's disease; PDGF, platelet‐derived growth factor; PI3K, phosphatidylinositol‐4,5‐bisphosphate 3‐kinase; PSD‐95, postsynaptic density protein 95; *PSEN*, Presenilin; pTau, phosphorylated Tau protein; rTMS, repetitive transcranial magnetic stimulation; s, seconds; SAM, senescence‐accelerated mice; sAPP, soluble APP; SNAP25, synaptosomal‐associated protein 25; SNc, substantia nigra pars compacta; SYN, synapsin; SYP: synaptophysin; T2DM, type 2 diabetes mellitus; TrkB, tropomyosin receptor kinase B; VaD, vascular dementia; VEGF, vascular endothelial growth factor.

## 5 CLINICAL APPLICATIONS IN AD PATIENTS

The promising evidence discussed above has encouraged the exploration of the therapeutic potential of rTMS in AD patients. In recent years, there has been a growing body of clinical research supporting the potential of rTMS as a therapeutic intervention for patients with mild‐to‐moderate AD. Indeed, a series of meta‐analyses have reported favorable effects on global cognition, memory, and functional independence following rTMS, especially when delivered over regions such as the dorsolateral prefrontal cortex (DLPFC), precuneus, and temporal lobe.^27^, ^29^, ^265^, ^266^, ^267^, ^268^, ^269^

Although these results are encouraging, some limitations persist. Indeed, most studies involve relatively small sample sizes, short follow‐up periods, and variable stimulation parameters, which restrict reproducibility and generalizability. Furthermore, the mid‐to‐long‐term sustainability of clinical gains remains uncertain, although preliminary data suggest that personalized maintenance protocols or periodic booster sessions may help prolong therapeutic effects.^20^, ^270^ Additionally, the intensive nature of rTMS protocols—often requiring daily sessions over several weeks—raises practical concerns regarding scalability and patient compliance, especially in more advanced stages of AD. To tackle these challenges, the development of home‐based protocols and simplified technologies, as has already been implemented in neurodegenerative and other types of neurological disorders, is being explored.^271^, ^272^, ^273^ Although not directly applicable to rTMS, such innovations could complement its use or support combined interventions. In parallel, combining rTMS with pharmacological agents or cognitive training represents an emerging strategy to enhance neuroplasticity and potentially achieve more durable outcomes.^144^, ^274^, ^275^, ^276^ Furthermore, while most studies have focused on cognitive endpoints, rTMS has also demonstrated benefits for behavioral and neuropsychiatric symptoms, particularly apathy—an area where pharmacological options are limited and which deserves further investigation.^277^

Therefore, the cumulative evidence from clinical trials highlights rTMS as a promising, safe, and increasingly refined therapeutic strategy for AD. Nevertheless, future large‐scale, multicenter trials are necessary to standardize protocols, establish optimal stimulation parameters, and clarify the long‐term efficacy and feasibility of integrating rTMS into routine clinical practice.^12^ In this sense, it is reasonable to assume that the evidence emerging from animal models will be crucial for enhancing the effectiveness of rTMS treatments in patient populations. Indeed, many of the neurobiological mechanisms modulated by rTMS in preclinical studies not only appear to be fundamentally involved in the physiopathology of AD in human patients but also, notably, are susceptible to modulation by rTMS in clinical populations.

Importantly, although most research in AD animal models has focused on impaired hippocampal LTP‐like mechanisms, a parallelism with the human cerebral cortex has been advanced. A seminal study showed that the impairment of neocortical plasticity correlated with functional deficits of NMDA glutamate receptors in both AD patients and *APP/PSEN1* transgenic mice.^278^, ^279^ Furthermore, the most characteristic and reproducible feature of AD in animal models—the disruption of LTP‐like plasticity mechanisms—has also been demonstrated in AD patients, regardless of the age of onset and even during stages preceding the conversion to full‐blown dementia.^19^, ^280^, ^281^ Consistently, while amyloid plaques were once regarded as the primary contributors to cognitive deficits, recent evidence indicates that oligomeric forms of Aβ are actually the main drivers of synaptic dysfunction, which aligns more closely with cognitive impairments.^11,^ ^282^, ^283^Indeed, although some theoretical frameworks question the etiological relevance of Aβ in the context of AD, a consistent body of literature supports the notion that it plays a pivotal role in regulating neuronal and network activity and may contribute to the dysfunction of AD‐related brain networks.^6^, ^284^, ^285^ Notably, rTMS has demonstrated significant potential to influence synaptic plasticity even in humans, effectively restoring LTP‐like mechanisms disrupted by pathological processes.^286^, ^287^ A recent study consistently examined the long‐term effects of HF‐rTMS (10 Hz, sequential application over the bilateral DLPFC, five times per week on separate days for 4 weeks) on plasma levels of matrix metalloproteinases (MMPs) and tissue inhibitors of metalloproteinases (TIMPs) in patients with mild cognitive impairment (MCI).^288^ Indeed, the role of MMPs in regulating synaptic structure and ensuring proper synaptic plasticity is well established, and interest in the clinical implications, particularly in the field of neurodegeneration, is rapidly growing.^289^ Over 6 months, patients showed significant reductions in plasma levels of MMP1, MMP9, and MMP10, along with increases in TIMP1 and TIMP2.^288^ These biochemical changes correlated with improvements in visuospatial abilities, confirming that rTMS may influence neuroinflammatory mechanisms and synaptic plasticity in neurodegenerative conditions from early stages. Moreover, the study underscores the potential of MMPs and TIMPs as biomarkers for monitoring the efficacy of rTMS in clinical settings. In addition, the effects of rTMS in terms of increased neurotrophic factors (e.g., BDNF) and increased expression of IEGs, which in turn lead to activation of the BDNF‐TrkB pathway, have been extensively confirmed also in humans.^290^, ^291^, ^292^ Indeed, HF‐rTMS increased plasma BDNF levels in both rats and humans.^69^ This is crucial, as it suggests that the pathophysiological insights gained from animal models may also apply to humans. Consequently, the therapeutic potential of rTMS observed in these models could similarly benefit patients.

Notably, a significant area of focus in recent clinical research has been the personalization of rTMS parameters. While some studies have adopted a “one‐size‐fits‐all” approach with predefined cortical targets, others have employed neuroimaging‐guided protocols using structural or functional MRI or EEG to customize stimulation to individual brain profiles.^267^ More recently, rTMS parameter personalization has been achieved by combining TMS with EEG, enabling the identification of the most effective stimulation site and intensity for each patient. This approach aims to directly activate neural responses in the targeted area, the precuneus, within the default mode network (DMN).^20^, ^270^

This is particularly significant given the prevailing understanding that neurodegenerative diseases progress by spreading through networks of interconnected regions, a concept known as the “network degeneration hypothesis.”^293^, ^294^ Evidence has shown that rTMS, when applied to a specific site, can produce effects in remote but interconnected regions within an affected brain network, aiding in restoring network connectivity.^295^ Consistently, evidence in healthy adults has shown that multiple rTMS sessions increase functional connectivity among distributed cortical hippocampal network regions, leading to improvements in associative memory performance.^296^ In MCI or dementia due to AD patients, there is a characteristic impairment of the DMN, a network of interconnected areas active during restful wakefulness when the mind is not engaged in demanding cognitive tasks and thoughts wander.^297^ Numerous studies have demonstrated that DMN dysfunction correlates with the severity of cognitive deficits, and the progression of its alterations can predict clinical conversion.^298^, ^299^ Notably, a recent study combining TMS with EEG to investigate the propagation of neuronal activity induced by magnetic pulses revealed localized hyperexcitability (consistent with observations in animal disease models) and a lack of signal propagation within the DMN.^300^ Intriguingly, these neurophysiological features were also associated with AD patients' structural and cognitive attributes. Notably, various rTMS protocols have been shown to modulate the DMN, particularly the hippocampal‐parietal memory subsystem, by targeting strategic sites within this network, such as the precuneus and other superolateral and medial‐parietal cortical regions, as well as the DLPFC.^21^, ^296^, ^301^, ^302^, ^303^, ^304^ Similarly, applying bilateral rTMS to the cerebellum, which has been implicated in the pathophysiology of AD by various lines of evidence, may improve cognitive performance and increase functional connectivity between the cerebellum and cortical areas involved in the DMN.^55^, ^305^, ^306^ Moreover, recent research in AD patients has highlighted specific electrophysiological signatures, such as altered microstate dynamics (i.e., transient, quasi‐stable EEG states or patterns that show characteristic alterations in neurodegenerative diseases), associated with HF‐rTMS‐induced cognitive improvement.^307^ This provides novel insights into the neurofunctional substrates of treatment efficacy.

Therefore, it is not surprising that personalized rTMS protocols have also yielded promising results. In this regard, a functional MRI personalized, hippocampal network–targeted HF‐rTMS (20 Hz, 5 days a week for 4 weeks) applied to the left parietal area enhanced cognitive and functional performance, particularly by the eighth week.^308^ However, improvements were evident as early as 4 weeks after the conclusion of rTMS, accompanied by increased functional connectivity between the hippocampus and precuneus. Similarly, a critical study investigated the effects of a tailored HF‐rTMS (20 Hz, 10 sessions) protocol in AD patients, targeting the specific location, at the left parietal cortex level, with the highest functional MRI‐based connectivity to the ipsilateral hippocampus.^309^ This research demonstrated not only an improvement in visual recognition memory functions but also their association with elevated peripheral BDNF levels, increased antioxidant capacity, and decreased oxidant enzyme activity—effects that have been well‐documented in animal models following rTMS treatments.

Importantly, the observed resting‐state functional connectivity changes suggest that these approaches not only enable personalized treatments but also provide insight into the reorganization of brain networks underlying cognitive improvements following NIBS protocols.^309^ Indeed, in a seminal clinical trial by our group, HF‐rTMS protocol (20 Hz, 10 sessions over the first 2 weeks followed by one session per week for 22 weeks) was applied to the precuneus by using neuronavigated TMS‐EEG to personalize the specific location of stimulation and the intensity of rTMS. The clinical trial showed that personalized rTMS effectively stabilized cognitive performance (i.e., prevented further cognitive deterioration), cortical excitability, and gamma rhythm expression in the frontal regions.^20^ Consistently, gamma rhythms are characteristically reduced in these patients, likely due to alterations in GABAergic circuits, as previously discussed in animal models.^310^ Furthermore, these results align with a study that employed a 40‐Hz rTMS protocol (i.e., the gamma band) three times per week over four consecutive weeks, involving daily sessions of 30 rTMS trials (15 targeting the left angular gyrus followed by 15 for the right angular gyrus) in patients diagnosed with probable AD.^311^ Specifically, this treatment not only halted gray matter volume loss but also improved both local and global functional integration, enhancing the information flow from the left posterior temporoparietal region to the frontal areas and strengthening the dynamic connectivity between the anterior and posterior brain regions. Interestingly, another study reported macro‐ and micro‐structural preservation of gray matter following personalized treatment. Indeed, rTMS also increased functional connectivity within the precuneus, suggesting that this protocol might have the potential to halt atrophy progression in the stimulated network.^23^ This is consistent with findings in animal studies, where rTMS has been well‐documented to exert not only functional but also structural effects, such as the synthesis of synaptic proteins. Additionally, a subgroup of patients from the original clinical trial received an extra 52 weeks of treatment, adhering to the same protocol: an intensive phase involving daily stimulation for 2 weeks, followed by a maintenance phase with weekly sessions for the next 50 weeks.^270^ The findings indicated that this extended HF‐rTMS regimen may help slow the progression of cognitive decline, impairment in activities of daily living, and behavioral disturbances in patients with mild‐to‐moderate AD. Thus, the therapeutic benefits of rTMS can potentially be prolonged over time with an appropriate maintenance strategy, paving the way for its long‐term use as a disease‐modifying intervention aimed at delaying the progression of AD and its associated cognitive deterioration. However, it is also relevant to note, surprisingly, that comparative analyses have revealed no clear advantage of personalized targeting over standardized approaches.^267^ This may be attributed to the limited number of personalized studies, the reliance on structural rather than functional biomarkers for target identification, and the inherently network‐wide effects of rTMS that may extend beyond the stimulated site.^12^ Further studies are required to improve the applications of personalization strategies in clinical research, while also addressing crucial aspects such as disease duration, genetic background, and specific features of rTMS protocols, along with the synergistic effects of combined pharmacological therapies involving drugs that enhance neuroplasticity, such as dopaminergic agonists.^274^

Notably, while it is plausible that both LF‐ and HF‐rTMS may exert therapeutic effects, likely differing depending on the stage of the disease and the genetic and pathophysiological background according to evidence from animal models, HF‐rTMS protocols seem to yield superior benefits compared to LF‐rTMS.^28^, ^312^ Indeed, a study that consistently compared the effects of LF (1 Hz) and HF‐rTMS (20 Hz) applied bilaterally to the DLPFC in AD patients indicated that the latter resulted in more significant improvements across all cognitive measures and functional independence at every time point at the end of treatment.^313^ The effects of HF‐rTMS are not confined to the CNS but also extend to the immune system.^69^ Specifically, this protocol appears to increase BDNF–TrkB signaling and TrkB–NMDAR interaction in neurons and lymphocytes as well. Consistently, through the TrkB pathway, BDNF promotes lymphocyte survival and differentiation.^314^, ^315^ Importantly, lymphocytes can cross the BBB and influence the CNS microenvironment by producing cytokines and chemokines that modulate inflammatory processes within the brain.^316^ This finding gains further importance, considering recent evidence highlighting the bidirectional communication between the immune system and the CNS, whose dysregulation has been implicated in the pathophysiology of numerous pathological conditions.^317^ Therefore, it seems reasonable that the induction of neuronal plasticity through rTMS may be associated with favorable immunomodulation, offering a dual benefit against neuroinflammatory processes which, as discussed in earlier sections, are central to the pathophysiology of AD.

While the efficacy of rTMS in enhancing the clearance of neurotoxic proteins has been robustly demonstrated in animal models (see subsection 4.5), evidence supporting similar effects in patients with AD remains limited. To bridge this gap, future studies will be essential in assessing the extent to which rTMS may promote the clearance of pathological proteins in humans. Moreover, although specific investigations in AD populations are still lacking, several techniques have been successfully employed to explore rTMS‐induced changes in brain function in both healthy individuals and clinical cohorts.^67^, ^318^, ^319^ For instance, positron emission tomography (PET) imaging using dopaminergic tracers has demonstrated that rTMS modulates dopaminergic neurotransmission in both healthy volunteers and patients with PD.^320^, ^321^ Given the growing recognition of dopaminergic dysfunction as a relevant pathophysiological mechanism in AD, it is plausible that similar PET‐based approaches could yield valuable insights into the neuromodulatory effects of rTMS in this context.^322^ Similarly, it will be essential to employ fluid biomarkers not only for diagnostic purposes but also as outcome measures of treatment efficacy.^323^ Given their well‐established role in the pathophysiology of AD, these biomarkers—particularly those derived from blood, which are more easily accessible—may offer valuable and practical tools for monitoring therapeutic impact, an aspect currently underrepresented in the existing literature.

Nonetheless, recent studies have begun to address this issue. In one investigation, serum levels of Aβ40, Aβ42, and total Aβ were significantly reduced at 3, 4, and 6 weeks of HF‐rTMS (20 Hz, 5 sessions per week over 6 weeks) in patients with AD.^324^ In contrast, serum levels of the soluble ectodomain of the p75 neurotrophin receptor (p75ECD)—known to inhibit Aβ aggregation and block Aβ‐induced neurotoxicity—increased progressively over the same treatment period.^325^, ^326^ Furthermore, Aβ serum levels were significantly and negatively correlated with cognitive and functional performance, while p75ECD levels showed a positive correlation.^324^ Finally, a recent study investigated the effects of rTMS treatment on AD patients, focusing on assessing hippocampal metabolites—particularly N‐acetyl aspartate—using a magnetic resonance spectroscopy protocol.^327^ Correlation analysis revealed that improved visual memory scores were significantly associated with increased N‐acetyl aspartate levels. This finding suggests a potential partial attenuation of the well‐documented cholinergic deficit in AD, especially considering that cholinergic circuitry is essential for the proper functioning of hippocampal synapses.^328^, ^329^ These findings not only support the therapeutic promise of rTMS but also underscore the value of in vivo metabolic markers in guiding personalized diagnostic and treatment strategies.

**FIGURE 1:**
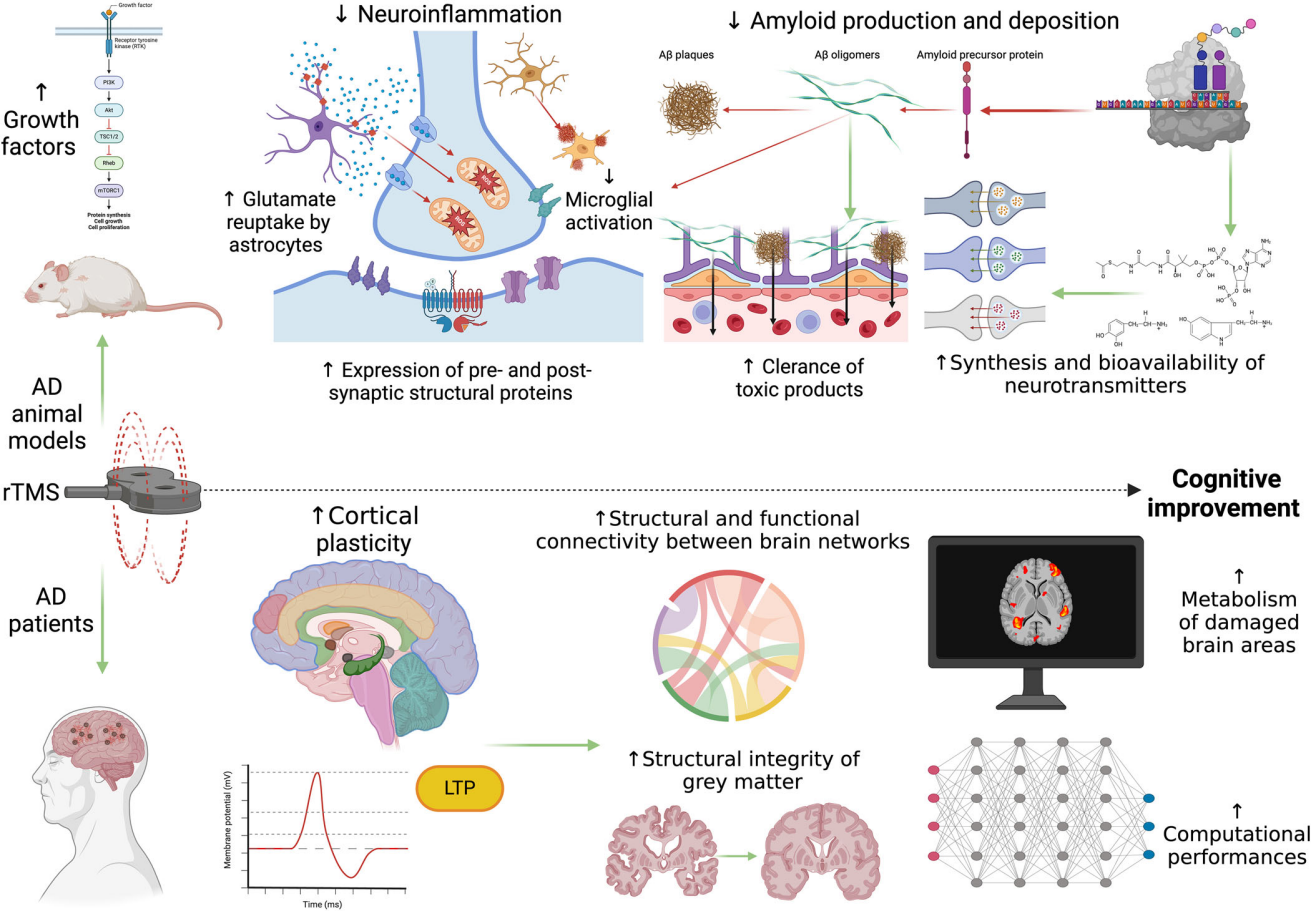
Mechanisms of rTMS‐induced neuroplasticity and therapeutic effects in AD. rTMS promotes neuroplasticity and cognitive improvement by modulating key pathological mechanisms in AD. Indeed, in animal models, rTMS enhances synaptic function by increasing the expression of structural synaptic proteins, reducing neuroinflammatory processes (e.g., by promoting glutamate reuptake and preventing microglial activation), upregulating growth factors, and increasing neurotransmitter synthesis. It also decreases amyloid production and deposition while facilitating the clearance of toxic aggregates, ultimately improving performance and behavior. Accordingly, by acting on these mechanisms, also in AD patients rTMS fosters cortical plasticity, restores LTP‐like mechanisms in key brain areas, strengthens structural and functional brain connectivity, and preserves the integrity of gray matter. These effects contribute to enhanced metabolism in damaged brain regions and improved cognitive functions, highlighting rTMS as a promising neuromodulatory intervention in AD patients. AD, Alzheimer's disease; LTP, long‐term potentiation; rTMS, repetitive transcranial magnetic stimulation. Created with BioRender.com.

Figure 1 provides a concise overview of the mechanisms underlying rTMS effects in animal models and the therapeutic benefits observed in AD patients.

### 5.1 Safety considerations about rTMS

As rTMS becomes increasingly integrated into both clinical practice and experimental research, safety remains a central concern. TMS is widely recognized as a safe and well‐tolerated technique, with the most commonly reported side effects being transient headaches or localized scalp discomfort.^330^ Moreover, although the risk of seizure induction is exceedingly rare, it can be further minimized by strictly adhering to established safety guidelines and comprehensively screening for contraindications, such as a history of seizures or the presence of cranial metal implants. This issue is particularly relevant in the context of AD patients, who, as previously discussed, often exhibit cortical hyperexcitability, especially in regions belonging to networks critically implicated in AD pathophysiology.^209^, ^300^ In such cases, concerns regarding HF stimulation protocols, which further increase cortical excitability, may be legitimate—even if the incidence of seizures is less than 1 in 60,000 and occurs particularly in cases of structural lesions such as strokes or brain tumors.^331^ This highlights the importance of adopting personalized approaches to treatment, for example, by applying HF‐rTMS to hypoexcitable areas and, conversely, using LF‐rTMS in the presence of cortical hyperexcitability. The integration of neurophysiological tools such as EEG or combined TMS‐EEG is likely to play a crucial role in guiding such individualized protocols.^20^

Importantly, a systematic review and meta‐analysis of 13 studies reported no seizures across all included trials.^27^ Another recent systematic review and meta‐analysis not only confirmed the absence of severe adverse events but also showed that only mild effects—such as headaches, fatigue, and scalp discomfort—were reported, which occurred in both active and sham stimulation groups.^29^ Furthermore, the safety and tolerability of rTMS have been further reinforced by findings from a recent large sample clinical trial, which demonstrated mild and infrequent adverse effects not only during the intensive treatment phase but also following an extended maintenance phase.^20^, ^270^

Finally, the safety of rTMS treatments has not only been demonstrated clinically but is also supported from a neurobiological perspective. Neurofilament light chains (NfL) are widely recognized as a non‐specific yet sensitive biomarker of neuroaxonal damage.^332^ Notably, several studies have shown that rTMS treatment—beyond yielding clinical improvements in conditions such as alcohol use disorder and postoperative delirium—has also been associated with a significant reduction in serum NfL levels.^333^, ^334^ This decrease may reflect a potential neuroprotective effect. Indeed, one study investigating this aspect also reported an increase in BDNF, whose role in reducing neuronal injury, promoting recovery, and facilitating neural regeneration has been thoroughly discussed in the previous sections.^334^ These findings are especially valuable as they offer neurobiological support for the clinical safety of rTMS and highlight shared mechanisms between preclinical models and patients undergoing treatment.

## 6 POTENTIAL LIMITATIONS AND CHALLENGES IN TRANSLATING PRECLINICAL EVIDENCE TO PATIENTS

Although the previous sections extensively discuss the shared neurobiological and pathophysiological mechanisms between AD animal models and human patients, along with promising preliminary evidence suggesting the potential for translating preclinical findings into more effective treatment strategies, several critical limitations and challenges require caution.

First, while preclinical evidence supports the efficacy of rTMS from a neurobiological perspective, translating these findings into consistent clinical benefits remains highly complex. Neurobiological differences likely play a key role in this regard. Indeed, animal models exhibit life cycles, metabolic processes, and neuroanatomical and physiological organization that often differ profoundly from those of humans.^335^, ^336^ These differences may lead to distinct responses to NIBS treatments despite the modulation of largely overlapping molecular pathways. For instance, in humans, it is possible to achieve relatively focal stimulation of specific neural targets without the confounding factors associated with the need for sedation, forced immobilization, or broader stimulation due to the considerably smaller cranial geometry of animal models. In contrast, these limitations are frequently present in preclinical studies, potentially influencing the effects of stimulation and complicating the generalization of findings to humans.^337^

Moreover, as previously discussed, animal models of disease are specifically designed to exhibit hallmark neuropathological alterations in an accelerated manner, often through the overexpression of genes implicated in AD pathogenesis or exposure to substances and conditions that induce advanced pathological states within a compressed timeframe.^90^ While these strategies have been instrumental in advancing our understanding of AD pathophysiology, it is crucial to recognize that, in humans, the disease typically manifests after decades of progressive damage, likely driven by multifactorial mechanisms involving multiple and concurrent pathological factors.^3^, ^338^ Consequently, more refined animal models that more faithfully replicate the human condition may be necessary before confirming the complete and effective translatability of preclinical findings.

Furthermore, in many cases, animals were sacrificed immediately after the completion of rTMS treatments to analyze the neurobiological changes induced by NIBS protocols. While this approach has provided detailed insights into the molecular pathways modulated by rTMS, it significantly limits the ability to determine the duration of these effects and, more importantly, their long‐term impact on the longitudinal trajectory of the disease. These challenges also persist in human studies, underscoring the need for future research with larger sample sizes and extended follow‐up periods to track the temporal evolution of rTMS‐associated benefits.^12^ Standardizing NIBS protocols across research laboratories and integrating results from multimodal sources, such as neurophysiological data, functional neuroimaging, and circulating biomarkers, could help address this limitation, resulting in more robust and reproducible findings.

In summary, while preclinical evidence is encouraging and suggests relevant applications given the neurobiological similarities between NIBS effects in humans and animal models, several unresolved challenges may limit its efficacy and translatability to AD patients. Nonetheless, optimizing disease models and refining rTMS protocols will likely help address these limitations, ultimately enhancing our understanding of AD pathophysiology and improving therapeutic strategies for its treatment.^12^

## 7 CONCLUSION

Taken together, the data reviewed here demonstrate that, although AD animal models are not yet qualified reproducing the heterogeneity and sequence of regional neuropathological changes observed in human patients, they have significantly supported and advanced our understanding of the pathophysiology of this complex disease. Many of the pathways characteristically altered in AD, including disruption of LTP‐like mechanisms in both hippocampal and cortical regions, neurotransmitter circuitry, excitation/inhibition balance, receptor and voltage‐gated channel expression, neurotrophic factors expression, and neuroinflammatory processes, are specifically modulated by rTMS treatments. Indeed, extensive research has shown that the neurobiological effects of rTMS in healthy animals are broad, and the specific modulation of these mechanisms in AD models is associated with significant improvements, not only at the neurobiological level but also, importantly, in behavioral and cognitive outcomes. These findings support the use of rTMS treatments in AD patients, as the pathophysiological pathways studied in animal models parallel, albeit with some limitations, to those observed in patients. Therefore, it is not surprising that these techniques are increasingly demonstrating promising data and results in clinical research settings. A deeper understanding of the neurobiological effects of rTMS is likely to further develop, refine, and optimize NIBS strategies based on the precision medicine framework. Considering that recent evidence suggests the possibility of identifying patients in the AD spectrum from the early stages through a noninvasive strategy, for example, through blood‐based biomarkers or, indeed, NIBS, it is likely that rTMS protocols might yield relevant clinical applications in the coming decades to slow disease progression from asymptomatic stages.^339^, ^340^, ^341^, ^342^ In this regard, animal models of AD will continue to play a pivotal role, particularly as new models are developed that more accurately replicate critical aspects of pathogenesis, perhaps in models involving species, such as cats and, most notably, non‐human primates, where rTMS can elicit effects that more closely mirror those observed in humans.

rTMS is a clear‐cut emerging example of precision medicine in neurology. In the near future, rTMS will likely be part of the evolving differentiated clinical care and treatment landscape with different treatments and combinations at specific stages, effectively targeting biomarkers and liquid biopsy‐identified mechanisms that will benefit the globally increasing population of AD patients.^343^ Further investigations will guide research toward increasingly personalized and effective treatment strategies for AD patients based on well‐defined mechanisms of action.

## AUTHOR CONTRIBUTIONS

Giacomo Koch conceived the manuscript. Annibale Antonioni and Giacomo Koch wrote the first draft of the manuscript. Alessandro Martorana, Emiliano Santarnecchi, and Harald Hampel edited the manuscript. All the authors validated and approved the final manuscript.

## CONFLICT OF INTEREST STATEMENT

Annibale Antonioni declares no conflicts of interest related to this manuscript. Giacomo Koch is scientific co‐founder and holds stocks of Sinaptica Therapeutics. Giacomo Koch has received payment or honoraria for lectures, presentations, speakers’ bureaus, manuscript writing, or educational events from Epitech, Roche, Novo Nordisk. Giacomo Koch and Alessandro Martorana have the following patent issued: Combination drug formulations including rotigotine and an acetylcholinesterase inhibitor for the treatment of neurodegenerative diseases (n. 20230381512); Giacomo Koch and Emiliano Santarnecchi have the following patent issued: Systems and methods for providing personalized targeted non‐invasive stimulation to a brain network (n. 11998740). GK reports grants from BrighFocus Foundation, Epitech, Alzheimer's Drug Discovery Foundation (ADDF), Italian Ministry of Health and non‐financial support from UCB Pharma outside the submitted work. Alessandro Martorana reports grants from Alzheimer's Drug Discovery Foundation, BrighFocus Foundation, Italian Ministry of Health and non‐financial support from UCB Pharma outside the submitted work.

Harald Hampel is an employee of Eisai Inc.; however, this article does not represent the opinion of Eisai. His contribution to this article reflects only and exclusively his academic and scientific expertise and was initiated during an academic appointment at Sorbonne University, Paris, France. He serves as a Reviewing Editor and previously as Senior Associate Editor for the journal Alzheimer's & Dementia, the journal of the Alzheimer's Association.

Harald Hampel is inventor of 11 patents and has received no royalties:

1. In Vitro Multiparameter Determination Method for The Diagnosis and Early Diagnosis of Neurodegenerative Disorders Patent Number: 8916388

2. In Vitro Procedure for Diagnosis and Early Diagnosis of Neurodegenerative Diseases Patent Number: 8298784

3. Neurodegenerative Markers for Psychiatric Conditions Publication Number: 20120196300

4. In Vitro Multiparameter Determination Method for The Diagnosis and Early Diagnosis of Neurodegenerative Disorders Publication Number: 20100062463

5. In Vitro Method for The Diagnosis and Early Diagnosis of Neurodegenerative Disorders Publication Number: 20100035286

6. In Vitro Procedure for Diagnosis and Early Diagnosis of Neurodegenerative Diseases Publication Number: 20090263822

7. In Vitro Method for The Diagnosis of Neurodegenerative Diseases Patent Number: 7547553

8. CSF Diagnostic in Vitro Method for Diagnosis of Dementias and Neuroinflammatory Diseases Publication Number: 20080206797

9. In Vitro Method for The Diagnosis of Neurodegenerative Diseases Publication Number: 20080199966

10. Neurodegenerative Markers for Psychiatric Conditions Publication Number: 20080131921

11. Method for diagnosis of dementias and neuroinflammatory diseases based on an increased level of procalcitonin in cerebrospinal fluid: Publication number: United States Patent 10921330. Author disclosures are available in the Supporting Information.

## ETHICS STATEMENT

Not applicable.

## Supporting information



Supporting Information

## Data Availability

All data used to write this review are derived from scientific publications listed in the References.
